# Technology Support Challenges and Recommendations for Adapting an Evidence-Based Exercise Program for Remote Delivery to Older Adults: Exploratory Mixed Methods Study

**DOI:** 10.2196/27645

**Published:** 2021-12-09

**Authors:** Nancy Gell, Elise Hoffman, Kushang Patel

**Affiliations:** 1 Department of Rehabilitation and Movement Science University of Vermont Burlington, VT United States; 2 Department of Anesthesiology and Pain Medicine University of Washington Seattle, WA United States

**Keywords:** tele-exercise, technology, older adults, adult learning theory, knee osteoarthritis, mobile phone

## Abstract

**Background:**

Tele-exercise has emerged as a means for older adults to participate in group exercise during the COVID-19 pandemic. However, little is known about the technology support needs of older adults for accessing tele-exercise.

**Objective:**

This study aims to examine the needs of older adults for transition to tele-exercise, identify barriers to and facilitators of tele-exercise uptake and continued participation, and describe technology support challenges and successes encountered by older adults starting tele-exercise.

**Methods:**

We used an exploratory, sequential mixed methods study design. Participants were older adults with symptomatic knee osteoarthritis (N=44) who started participating in a remotely delivered program called Enhance Fitness. Before the start of the classes, a subsample of the participants (n=10) completed semistructured phone interviews about their technology support needs and the barriers to and facilitators for technology adoption. All of the participants completed the surveys including the Senior Technology Acceptance Model scale and a technology needs assessment. The study team recorded the technology challenges encountered and the attendance rates for 48 sessions delivered over 16 weeks.

**Results:**

Four themes emerged from the interviews: participants desire features in a tele-exercise program that foster accountability, direct access to helpful people who can troubleshoot and provide guidance with technology is important, opportunities to participate in high-value activities motivate willingness to persevere through the technology concerns, and belief in the ability to learn new things supersedes technology-related frustration. Among the participants in the tele-exercise classes (mean age 74, SD 6.3 years; 38/44, 86% female; mean 2.5, SD 0.9 chronic conditions), 71% (31/44) had a computer with a webcam, but 41% (18/44) had little or no experience with videoconferencing. The initial technology orientation sessions lasted on average 19.3 (SD 10.3) minutes, and 24% (11/44) required a follow-up assistance call. During the first 2 weeks of tele-exercise, 47% of participants (21/44) required technical assistance, which decreased to 12% (5/44) during weeks 3 to 16. The median attendance was 100% for the first 6 sessions and 93% for the subsequent 42 sessions.

**Conclusions:**

With appropriate support, older adults can successfully participate in tele-exercise. Recommendations include individualized technology orientation sessions, experiential learning, and availability of standby technical assistance, particularly during the first 2 weeks of classes. Continued development of best practices in this area may allow previously hard-to-reach populations of older adults to participate in health-enhancing, evidence-based exercise programs.

## Introduction

### Background

Most older adults in the United States live with a chronic disease, such as knee osteoarthritis, with an estimated 62% to 67% reporting multiple chronic conditions [1,2]. Exercise reduces the risk of at least 25 chronic diseases by 20% to 30% and is associated with improved quality of life and physical and cognitive function in older adults [3-5]. Despite the benefits of exercise, participation is low among older adults, with relatively few older adults with chronic conditions meeting the recommendations for exercise [6-8].

Multiple community-based exercise programs, including Enhance Fitness, Fit & Strong!, Active Living Everyday, and Geri-Fit, have been developed to address the strength, balance, and physical fitness of older adults [9]. Benefits from participation in these programs include improvements in physical performance, aerobic endurance, self-efficacy for exercise, self-rated health, and decreased pain [10-12]. Community-based classes have also demonstrated reduced loneliness and social isolation among older adults [13]. To promote participation among older adults, free access to community-based exercise is offered as a benefit through some Medicare Advantage Plans, such as Silver Sneakers and Silver&Fit, and health maintenance organizations, including Kaiser Permanente. However, in March 2020, most community-based programs serving older adults ceased or reduced their capacity, to comply with physical distancing mandates to prevent the spread of COVID-19.

Tele-exercise, including remote delivery of exercise classes through videoconferencing technology, has emerged as a means for community-based programs to resume guided exercise sessions while complying with the physical distancing restrictions during the COVID-19 pandemic [14]. The Gerofit-to-Home program has demonstrated preliminary evidence that older veterans who transition from facility-based to remotely delivered programs retain their physical function [15]. Previous studies have also shown the feasibility and acceptability of videoconference-delivered tai chi and yoga classes for older adults [16-18]. However, all 3 studies used proprietary technology that required specialized equipment installation at the participants’ homes. In addition, the technology did not allow the participants to see or interact with other participants in the exercise sessions, thereby reducing the social support asset of group exercise. With increasing ownership of devices with audiovisual capability, such as laptop computers, tablets, and smartphones [19], many older adults have access to videoconferencing technology without the need for proprietary equipment. However, little is known about the technology support needs of older adults to facilitate participation in tele-exercise classes.

### Objectives

The adaptation to remote delivery of exercise classes through videoconferencing (referred to as tele-exercise) is needed to meet the needs of older adults abiding by the physical distancing recommendations. In addition, expanding tele-exercise options increases the potential to include older adults who previously had limited access to community-based programs due to rural residence, caregiving responsibilities, transportation, and other challenges [20-22]. Understanding the technology support needs of older adults is critical to foster the success with tele-exercise participation. Therefore, we aim to (1) examine the needs of older adults previously enrolled in community-based exercise for transition to tele-exercise, (2) identify barriers to and facilitators of tele-exercise uptake and continued participation, and (3) describe technology support challenges and successes encountered by older adults starting tele-exercise.

## Methods

### Methodological Approach and Study Design

We used an exploratory, sequential mixed-methods design in which qualitative data were initially collected and analyzed. This approach allowed for input from older adult participants that was then used to inform the delivery of remote Enhance Fitness and the preparation for technical support before and during the remote classes. The rationale for using a mixed-methods approach was to develop a more comprehensive understanding of the technology needs and barriers related to remote exercise participation among older adults [23]. The qualitative data examined the older adults’ perspectives on technology adoption and needs in transitioning from in-person to tele-exercise. The findings then informed the collection of quantitative data that were used to enumerate the technical support needs and determine the extent to which these needs were addressed in the tele-exercise program. Subsequently, the qualitative and quantitative findings were integrated for interpretation [24], wherein we considered the quantitative results in the context of the qualitative findings. The Standards for Reporting Qualitative Research were used to guide the reporting of the study results and methods [25]. All the procedures were approved by the University of Washington Institutional Review Board, and informed consent was obtained from all the participants.

### Participants and Recruitment

**Figure 1:**
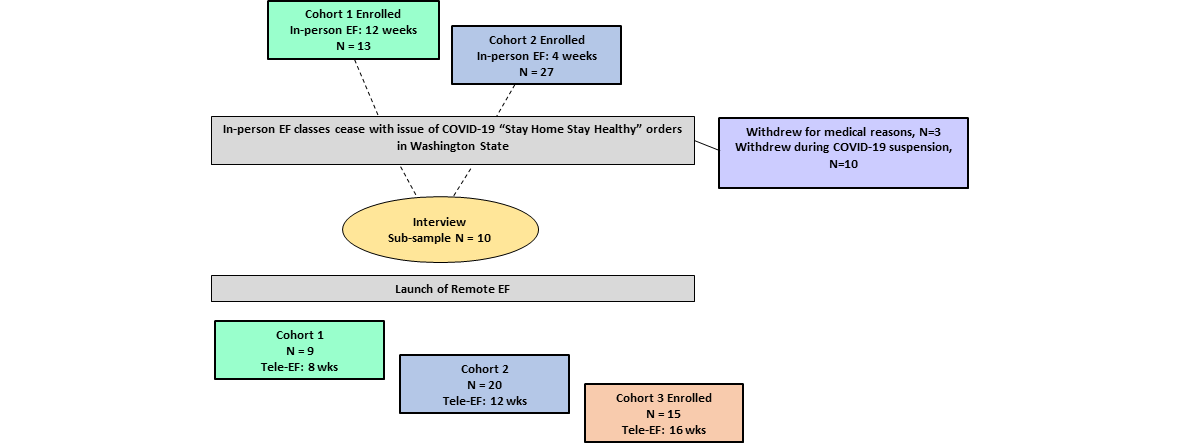
Participant flow diagram. EF: Enhance Fitness.

Participants were recruited from communities in and around Seattle, Washington, using a multimodal approach, including mailing letters and brochures to the UW Medicine patients and posting on social media. Between October 2019 and September 2020, participants were enrolled in a randomized controlled trial (NCT04099394) comparing the combination of group exercise with either a group-based cognitive behavioral skills training program or a group-based health education program. The exercise program, Enhance Fitness, is an evidence-based and nationally disseminated program that involves instructor-led strength, endurance, and balance training for 1 hour, 3 days a week [26,27]. The Centers for Disease Control and Prevention recommend Enhance Fitness for arthritis management and the National Council on Aging recommends it for falls prevention [28,29]. All the participants were English-speaking, community-dwelling older adults (age ≥65 years) with symptomatic knee osteoarthritis and without cognitive impairment. Two-thirds of the participants (n=29) were enrolled in the trial before the COVID-19 pandemic halted all in-person Enhance Fitness exercise classes in Seattle, King County, Washington [30]. The participant flow diagram is shown in Figure 1. The participants were invited to participate in phone interviews about their technology support needs to transition to tele–Enhance Fitness classes. We used purposeful sampling to ensure interviews were conducted with the participants with a range of technology and computing experience, based on a questionnaire assessing technology ownership and history of technology use. The interviews were conducted until saturation was achieved, that is, until little to no additional information was emerging from conducting additional interviews. After the virtually delivered protocol of tele–Enhance Fitness was developed by the study team (KP and EH in consultation with NG and Ms Paige Denison, Enhance Fitness National Program Director), guided by the input gathered from the participant interviews and approved by the University of Washington Institutional Review Board, the trial’s Data Safety Monitoring Board, and the National Institute on Aging of the National Institutes of Health, the tele–Enhance Fitness classes began in July 2020. An additional 15 participants who did not have prior experience with in-person Enhance Fitness began the tele–Enhance Fitness classes in September 2020. The data reported in this study were collected between October 2019 and January 2021.

### Measures

#### Qualitative

The participants were interviewed through phone by 1 research team member (interviewer EH) who had prior training and experience in conducting interviews. The interviewer used a semistructured guide with open-ended questions and follow-up probes as needed (refer to Multimedia Appendix 1 for the interview guide). The interviews lasted an average of 27 (SD 9) minutes and included questions on (1) concerns about transitioning from in-person to remote exercise classes, (2) past experience with technology adoption, and (3) barriers to and facilitators of new technology adoption. All the interviews were audio-recorded and transcribed verbatim.

#### Quantitative

As part of the trial’s baseline assessment, the participants completed a series of questionnaires on demographic and health characteristics shown in Table 1. These data were collected through in-person interviews for cohorts 1 and 2, whereas cohort 3 completed the questionnaires on the web using REDCap (Research Electronic Data Capture; Vanderbilt University) electronic capture tools [31]. Physical activity was measured using a thigh-worn activPAL3 microaccelerometer (PAL Technologies) device that assessed the steps per day, averaged over the course of a week before the study interventions began. In addition, before beginning the tele–Enhance Fitness classes, all the participants completed a technology needs assessment and a survey questionnaire on technology use and acceptance. The technology use assessment adapted questions from the National Health and Aging Trends Study [32] and the Pew Research Center [33]. The 14-item short version of the Senior Technology Acceptance Model (STAM) was also collected, which has demonstrated reliability and validity for measuring (1) attitudinal beliefs, related to positive or negative feelings toward using technology; (2) control beliefs, a reflection of self-efficacy, facilitating conditions, and ease of use; and (3) anxiety in older adults, defined as apprehension about using technology [34]. Finally, as part of the trial protocol, the study team conducted one-on-one technology orientation meetings through Zoom (Zoom Video Communications) with the participants to orient them to the tele–Enhance Fitness program, including guided instruction on videoconferencing. When necessary, the study team provided instructions on accessing and using Zoom, through telephone before the orientation call. The study team systematically recorded the duration of the video call and all the challenges that the participants faced while using technology. Similarly, the study team also recorded the technology challenges encountered when the participants engaged in tele–Enhance Fitness classes and if telephonic assistance was necessary, the length of the call was recorded. Attendance rates were documented for all the classes. The study team members who conducted the orientation meetings and assisted the participants with technology challenges during the tele–Enhance Fitness classes were recent public health graduates from the University of Washington (including EH). These team members were trained in communicating effectively with older adults and were aware of the findings from the qualitative interviews.

**Table 1:** Participant characteristics.

Characteristic			Total sample (N=44)		Subsample with qualitative interviews (n=10)
Age (years), mean (SD)			74 (6.3)		76.5 (8.1)
**Sex, n (%)**					
	Women	38 (86)		9 (90)	
**Race and ethnicity, n (%)**					
	White	38 (86)		9 (90)	
	Black	3 (7)		1 (10)	
	Hispanic	1 (2)		0 (0)	
	Other	2 (5)		0 (0)	
**Education, n (%)**					
	High school graduate	1 (2)		0 (0)	
	Some college or vocational	7 (16)		2 (20)	
	College graduate	18 (41)		5 (50)	
	Masters or higher degree	18 (41)		3 (30)	
**Smoking history, n (%)**					
	Never smoked	25 (57)		6 (60)	
	Former smoker	19 (43)		4 (40)	
	Current smoker	0 (0)		0 (0)	
Total number of medical conditions, mean (SD)			2.5 (0.9)		2.6 (1.0)
Steps per day, mean (SD)			4516 (1437)		4201 (1315)
Lives alone, n (%)			24 (55)		6 (60)

### Tele–Enhance Fitness Delivery

After the individual technology orientation sessions were completed, the tele–Enhance Fitness classes were delivered 3 days per week for 1 hour over Zoom. The participants were provided with a link to the tele–Enhance Fitness classes through email. The Zoom session opened 5 minutes before the start of the class to allow socialization and opportunities to ask questions to the instructor. The Enhance Fitness-certified instructor led the exercise classes in accordance with the Enhance Fitness guidelines, including instructions for modifications to increase or decrease the level of difficulty. Each 1-hour–long tele–Enhance Fitness class included approximately 5 minutes of warm-up exercise, 20 minutes of aerobic exercise, 20 minutes of strength training, and 10-15 minutes of balance and stretching exercise. A research assistant attended all the web-based classes to provide technical support and monitor the safety and level of effort by the participants.

### Data Analysis

#### Qualitative

The interviews were independently coded by 2 members of the research team (NG and EH). The primary coder (EH) was the same individual who conducted the interviews. She has training in public health and received additional training and supervision from investigators with experience in using qualitative methods. The second coder (NG) has experience in the public health, including training and experience in using qualitative methods. Thematic analysis followed the guidelines described by Braun and Clark [35] to identify the themes related to older adults’ perspectives on technology needs for transitioning to tele-exercise. Both the coders independently read all the transcripts multiple times to identify the codes. Then, they discussed the inconsistencies in the coding until an agreement was reached. After the discussion, the codes were classified into 4 main themes. While reviewing the themes, the research team discussed the meaning behind the themes in relation to the study. The coding and analyses were conducted using Microsoft Word and Microsoft Excel.

#### Quantitative

Descriptive statistics were computed for all variables. We used the Kruskal-Wallis equality-of-populations rank test (ie, a nonparametric one-way analysis of variance) to determine whether the distribution of STAM subscales varied according to self-rated confidence to go on the web (Table 2). This test was used because the responses to the STAM were skewed. In Table 3, we compute the prevalence and incidence rates of the technology challenges encountered during the initial orientation call and the subsequent tele–Enhance Fitness classes, respectively. Prevalence rate was the number of participants who experienced a given technological challenge during the orientation call divided by the total number of participants (N=44). In contrast, the incidence rate for a specific challenge was calculated by dividing the number of times the challenge occurred by the total number of tele–Enhance Fitness classes attended by the participants. To facilitate interpretation, this quantity was then multiplied by 100 to reflect the number of times a challenge was encountered for every 100 people who attended a tele–Enhance Fitness class. Incidence rates are useful to report because they capture not only the single occurrence of a challenge experienced by a participant but also the recurrence of a challenge experienced by a participant (eg, forgetting to join with Zoom audio on a tablet) and account for different rates of tele–Enhance Fitness class attendance across the participants. All the statistical analyses were conducted using Stata SE 15.

**Table 2:** Senior Technology Acceptance Model (STAM) subscale scores according to self-rated confidence to go on the web.

STAM subscales	Total sample (N=44), median (IQR)	Not at all or only a little confident (n=6), median (IQR)	Somewhat confident (n=17), median (IQR)	Very confident (n=21), median (IQR)	*P* value
Attitudinal beliefs	8.7 (3)	5.0 (2.3)	8.3 (1.7)	10 (1.3)	<.001
Control beliefs	8.9 (2.5)	6.9 (2.3)	8.8 (1.8)	10 (1)	<.001
Gerontechnology anxiety	3.5 (4)	3.8 (2.5)	5.5 (3)	2 (4)	.046
Health	8.4 (1.6)	8.2 (1.2)	8.4 (1.6)	8 (1.6)	.85

**Table 3:** Challenges encountered implementing tele–Enhance Fitness at different phases of the program.

Challenges encountered			Initial orientation call (n=42), (setup phase)				First 2 weeks of tele–Enhance Fitness classes				Subsequent 2-month period of tele–Enhance Fitness classes		
			Value, n		Prevalence (%)	Value, n			Rate per 100 persons (Enhance Fitness sessions^a^)	Value, n			Rate per 100 persons (Enhance Fitness sessions^a^)
**Hardware or internet setup issues**													
	No device camera	1		2		0		0		0		0.0	
	No device microphone	1		2		0		0		0		0.0	
	Screen too small	0		0		2		0.9		0		0.0	
	Internet connectivity issues	0		0		3		1.4		5		0.9	
	Unable to log into email	2		5		0		0		0		0.0	
	Allowing Zoom camera access	1		2		0		0		0		0.0	
	Turning on computer sound	1		2		0		0		0		0.0	
**Physical setup**													
	Assembling wide-frame lens	9		21		0		0		0		0	
	Positioning lens on camera	13		31		1		0.5		0		0	
	Locating the front camera on device	5		12		0		0		0		0	
	Tablet or computer camera view	13		31		1		0.5		3		0.5	
	Room space	7		17		2		0.9		0		0	
	Backlit image	1		2		1		0.5		0		0	
**Zoom controls**													
	Trouble downloading Zoom	2		5		0		0		0		0	
	Joining or staying on Zoom meeting	0		0		22		10		15		2.6	
	Joining with Zoom audio	4		10		9		5.1		11		1.9	
	Using Zoom control buttons (ie, mute)	5		12		3		1.4		3		0.5	
	Switching to speaker or gallery view	9		21		0		0		1		0.2	
	Video turned off	0		0		2		0.9		0		0	
**Technology communication**													
	Lack of computer or tablet knowledge	2		5		2		0.9		0		0	
	Lack of understanding of technology terms	5		12		2		0.9		0		0	

^a^This incidence rate reflects the number of times a challenge is encountered for every 100 people who take a tele–Enhance Fitness class.

## Results

### Study Sample

The participants’ characteristics are shown in Table 1. The age distribution ranged from 66-92 (mean age 74, SD 6.3) years and 41% (18/44) of participants were aged ≥75 years. The study sample primarily comprised White women. Most participants had a college or graduate degree and approximately half of them (24/44, 55%) lived alone. Consistent with the epidemiology of the target population, the mean number of medical conditions was 2.5 (SD 0.9) with 67% (29/44) of participants having ≥3 conditions (Table 1). Finally, the participants walked an average of 4516 (SD 1437) steps per day, with 21% (9/44) walking <3000 steps per day. In general, the subsample of participants who were selected for the qualitative interviews had characteristics similar to those of the total study sample (Table 1).

### Interview Themes

#### Overview

We identified 4 themes related to the technology experience and needs for participation in tele-exercise: (1) participants desire features in a tele-exercise program that foster accountability, (2) importance of direct access to helpful people who can troubleshoot and provide guidance with technology, (3) opportunities to participate in high-value activities motivate willingness to persevere through the technology concerns, and (4) belief in the ability to learn new things supersedes technology-related frustration.

#### Theme 1: Participants Desire Features in a Tele-Exercise Program That Foster Accountability

Participants noted that a key value of community-based (in-person) classes was the accountability inherent in having a scheduled class with the same group of people each week:

I think one of the reasons I did well or that I managed with the exercise is I felt obliged to because everybody was doing it.

Having something that is scheduled, do it now three days a week, is going to get me to do it more...I'm probably more likely to do it. Well, actually it's the same as going to the exercise classes at the senior center.

The opportunity to socialize and *check-in* with other participants enhanced their desire to attend the exercise classes, which they believed should be duplicated with a remotely delivered class:

I'm assuming that doing this electronically or however it's going to be done, that there would be a chance to say, “Wait a minute, am I doing this right?”...So that kind of interaction. And ensure the joking and joshing with the other participants, just being friendly and interacting with them.

In addition, the accountability of a scheduled, live-streamed class versus *on-demand* videos was considered an asset by the participants. Adequate screen size was also noted as important for accountability to enable participants to see others in the class while also being seen by the instructor. Overall, the participants valued the accountability aspect inherent in the group classes and therefore recommended retaining the accountability features in the tele-exercise classes.

#### Theme 2: Direct Access to Helpful People Who Can Troubleshoot and Provide Guidance With Technology Is Important

The participants identified attributes of technical assistance critical to the successful transition to web-based exercise classes. One-on-one or small group instruction for the initial setup of technology was identified as key to a successful start. Some people wanted latitude to conduct the technical setup themselves, with guidance, rather than someone else doing it for them. Similarly, there was little consensus on the mode of instructions (ie, printed vs web-based video) with more importance placed on access to a person able to answer questions as they arise during the initial setup:

To be accessible without going through a hundred people to get to you to find out what it is I need help with. Your accessibility would be the most important thing to me.

The temperament of the person providing assistance was also noted as essential, as the participants clearly expressed the importance of avoiding frustration from the person providing technical assistance. Participants also stated a desire for affirmation that their technical challenges were not unique and that they, like other people in a similar situation, were capable of overcoming the challenge:

Let me know that I'm not the only one they're happening to. And let me know that I'm not going to lose the marvelous experience because of this problem...I mean, if someone was impatient with me or if something were verbalized like, “You're the only one that has these horrible problems,” that would be very discouraging.

The participants recognized the risk of personal frustration with using technology to access the exercise classes and felt that the attitude and patience of the person providing technical support would help to mitigate their frustration.

#### Theme 3: Opportunities to Participate in High-Value Activities Motivate Willingness to Persevere Through Technology Concerns

The participants acknowledged concerns about learning and using technology to access exercise classes. Reasons for concern ranged from previous challenges with adopting new technology to the perception that they might take action that resulted in irrevocable loss of software or files on the device. However, the participants stated that the access to guided exercise was sufficiently important to work through the technology-related challenges that arise while joining tele-exercise classes. They noted that the benefit they experienced with the in-person exercise classes motivated them to overcome the technology challenges to participate in the exercise classes again, although remotely:

In other words, I am not adept at computer issues. I think that because I really want to do this...I believe that being able to do this stuff again is going to be so great that I’ll be able to get enough help and guidance. So it won't be as tough as I anticipate it might be, but there are things to overcome and there are things to learn and so on.

I'm not eager to, but I will if that's what we need to do...

Overall, the participants were realistic about the likely barriers to initiating a new activity dependent on learning and using technology. Given their past experience with the group exercise classes, they prioritized the benefits of resuming exercise over the barriers of accessing it through technology.

#### Theme 4: Belief in the Ability to Learn New Things Supersedes Technology-Related Frustration

Some participants reported previous positive experiences with using technology for communication and videoconference activities, particularly since the start of the COVID-19 pandemic. However, overall, the participants acknowledged the likelihood of experiencing frustration in accessing remote exercise classes. Notably, the participants also believed in their ability to learn new technology and overcome challenges:

Learning beforehand instead of learning as we go. I think that would be very helpful. I am a good learner. I still have good intelligence and good ability to pay attention, and so I have good learning skills. That's a very good thing that's still with me.

So since I've been retired, I've tried to figure out things on my own and I'm making some progress on some things, but I tend to have sort of a short, short temper on it. It's like, I can't believe this. Who needs it anyway? And then after a few days, I think well I need to stay up with the modern age. Surely I could do this.

It was important to the participants that they be perceived as capable of adapting to the circumstances, to mirror their own beliefs in their abilities to work with technology. Although technology-related frustration was acknowledged, it was not considered an insurmountable impediment because of their belief in their own abilities and the perceived importance of participating in exercise.

### Technology Use and Acceptance

Most participants in the tele–Enhance Fitness classes (40/44, 91%) owned a smartphone and had broadband at home, but approximately one-third did not own a tablet and one-third did not own a computer with a webcam (Table 4). Notably, of those who did not own a computer with a webcam, 69% (31/44) owned a tablet that they could use for tele-exercise (cross-tabulation not shown in Table 4). In addition, 80% (35/44) reported using an app in the last month and 84% (37/44) reported emailing and texting on most days in the last month. In contrast, only 9% (4/44) used a videoconferencing platform on most days in the last month and 41% (18/44) reported either no or rare videoconferencing. Approximately half of the participants (21/44, 48%) were very confident in their ability to use a device to go on the web (Table 4), but 14% (6/44) reported being not at all or only a little confident to go on the web.

Table 2 presents the STAM subscale results for the total sample and is stratified by self-rated confidence to go on the web. Attitudinal and control beliefs were generally high in the total sample, indicating high levels of perceived usefulness of technology in daily life (attitudinal beliefs) and confidence in using technology (control beliefs). In addition, anxiety about using technology (*gerontechnology anxiety*) was generally mild in the overall population; however, there was substantial variation in anxiety scores as the SD was large. In fact, attitudinal and control beliefs and anxiety varied significantly according to self-rated confidence to go on the web (Table 2); the participants with less confidence had lower attitudinal and control belief scores. Notably, the participants who were somewhat confident in their ability to go on the web had the highest levels of anxiety about using technology.

**Table 4:** Technology ownership and use survey (N=44).

Characteristic			Value, n (%)
**Owns a mobile phone**			
	No	3 (7)	
	Yes, a cellphone	1 (2)	
	Yes, a smartphone	40 (91)	
Uses home broadband			42 (93)
**Owns a computer**			
	No	4 (9)	
	Yes, but does not use it	3 (7)	
	Yes, but it does not have a webcam	6 (14)	
	Yes, and it has a webcam	31 (71)	
**Owns a tablet**			
	No	13 (30)	
	Yes	30 (68)	
	Yes, but does not know how to use it	1 (2)	
**Used an app on cellphone or tablet in the last month**			
	No	6 (14)	
	Yes	35 (80)	
	Not sure	3 (7)	
**Messaged someone in the last month**			
	Yes, emailed and texted	40 (91)	
	Yes, emailed but did not text	3 (7)	
	Yes, texted but did not email	1 (2)	
**Frequency of messaging in the last month**			
	Rarely	1 (2)	
	Somedays	6 (14)	
	Most days	37 (84)	
**Used a videoconference platform in the last month**			
	No	10 (23)	
	Yes, but rarely	8 (18)	
	Yes, on somedays	22 (50)	
	Yes, on most days	4 (9)	
**Overall confidence using digital or electronic devices to go on the web**			
	Not at all confident	1 (2)	
	Only a little confident	5 (11)	
	Somewhat confident	17 (39)	
	Very confident	21 (48)	

### Technology Support Calls for Tele–Enhance Fitness

Nine participants attended a phone call (mean time of 14 minutes, SD 7 minutes) for guidance on accessing and opening Zoom for the orientation meeting. Subsequently, 42 participants completed the initial technology orientation by videoconference through Zoom. The orientation included an overview of the tele–Enhance Fitness program, orientation to the videoconference platform, and a safety check to confirm the availability of a stable chair of standard height and a clear 5×5 foot space to exercise in. The duration of the orientation ranged from 5 to 45 (mean 19.3, SD 10.3) minutes. A second follow-up Zoom meeting was completed with 2 participants who needed additional training support. These second calls lasted for 10-20 (mean 15, SD 7.1) minutes. Two participants declined the orientation meeting, citing familiarity with using Zoom from previous experience.

During the first 2 weeks of tele–Enhance Fitness classes, approximately half (21/44, 48%) of the participants required a single telephone call to address the technology challenges encountered while engaging in tele–Enhance Fitness classes. The duration of these support calls ranged from 2 to 15 (mean 6, SD 3.3) minutes. Of the 21 participants who required additional support, only 2 (10%) required a second call during the initial 2-week period. Subsequently, after participating in tele–Enhance Fitness for 2 weeks, 11% (5/44) of participants needed a support call once and 5% (2/44) of participants required assistance 2 or more times. In contrast to the first 2 weeks of classes when there were 24 calls (mean duration 6.5, SD 3.6 minutes), there were 11 calls (mean duration 5.6, SD 3.0 minutes) in the subsequent 2-month period of tele–Enhance Fitness classes. Thus, the need for technology support for older adults participating in tele–Enhance Fitness decreased substantially after the first 2 weeks of classes.

### Technology Challenges Encountered

Three participants who did not have sufficient broadband access at home were given cellular-enabled tablets that were sponsored by the study. Table 3 presents the rates of the challenges encountered during the different stages of implementing tele–Enhance Fitness. A common challenge for participants was assembling and placing a clip-on magnifying lens over the webcam, which enabled the exercise instructor to observe the participant’s full body when exercising at a safe distance from the device. Other common challenges addressed during the initial technology orientation were navigating the camera’s view and learning how to use Zoom functions, including switching from speaker to gallery view. Cluttered room space was addressed by 17% (7/42) of the participants. Finally, limited knowledge of common technology use terms (eg, *scroll up/down* and swipe) hampered communication in 12% (5/42) of participants during the initial orientation. Once tele–Enhance Fitness classes began, difficulty in joining the Zoom meeting and not joining with Zoom audio were common in the first 2 weeks, but the incidence of these challenges decreased by 75% and 59%, respectively, over the subsequent 2-month period.

### Attendance

During the first 2 weeks of tele–Enhance Fitness (ie, 6 classes), the median attendance was 100% with an IQR of 83% to 100%. The median attendance for classes during weeks 3 to 16 was 93% (IQR 88%-98%). Interestingly, the overall attendance to tele–Enhance Fitness was better among the participants who did not have prior experience with in-person Enhance Fitness (cohort 3: median 96%, IQR 91%-98%) than among those who did (cohorts 1 and 2: median 92%, IQR 83%-96%).

## Discussion

### Principal Findings

This study provides insights into the technology support needs of older adults starting with tele-exercise classes. Key findings related to participants’ technical support needs, which were identified during the interviews, were incorporated into the technical orientation sessions. Overall, the interviews identified strong interest in technology use, motivated by the opportunity to participate in group exercise and targeted recommendations for a successful transition to tele–Enhance Fitness from the perspective of older adults. The high attendance rates throughout the 16-week tele–Enhance Fitness program, the experience of successfully addressing all technical challenges as they presented, and the diminishing technical challenges after 2 weeks of classes indicate successful transition to tele–Enhance Fitness for older adults.

Previous studies have found relatively positive attitudes among older adults toward technology when it supports desired activities and contains useful features [36,37]. We obtained a similar finding as evidenced by the theme of willingness to persevere through the technology challenges to participate in high-value activities such as exercise. With physical distancing preventing the participation in community-based (in-person) classes, the need to learn videoconference technology is currently relevant for attending livestream exercise classes. This theme also aligns with a key principle of motivational models in adult learning theory, which posits that learning is motivated by the relevance and impact to the learner [38,39]. To date, few studies have considered remote exercise options for older adults, perhaps due to the lower rates of technology use in older adults than in other generations [40]. However, in light of the findings of this study, the perceived challenges of technology adoption should be considered in relation to the value of the end purpose (eg, opportunities to participate in health-enhancing activities) in older adults.

Recommendations for adult education, based on social theories within adult learning theory [41,42], include creating an environment of cooperation and working collaboratively, which aligns with our themes of accountability and desire for direct access to helpful people. The participants articulated that a cooperative environment characterized by minimizing judgment or criticism is critical. This theme is also supported by previous research conducted in rural older adults, where motivation and interest to use technology was dependent on sufficient support and infrastructure [43]. A study of teaching methods for incorporation of technology in daily life for older adults noted the need to account for the learning processes of older adults, including flexible instructors who are able to respond to the unique learning situations and needs of older adults [44]. These findings suggest the need to consider how technical support is designed and delivered in transitioning to remotely delivered exercise classes, including communication skills for technology support providers and awareness of how their responses will impact the older adults they are teaching.

We did not find a consensus on how to provide the initial technology instructions (eg, step-by-step guidance as video instructions or written instructions). Prior work has demonstrated that the preferences for learning vary, depending on previous experience and knowledge, but also for training on specific tasks versus general tasks [45]. As noted in a study examining the approaches to training older adults to use technology, training should include a combination of procedures that include step-by-step guidance, with latitude for attention training, wherein participants decide on the steps but are given assistance as needed, dependent on individual factors [46]. Adult learning theory posits 2 stipulations: first, that adult learners build on the accumulation of life experience to aid learning, and second, instructions should account for variation in previous experience [38]. Our findings fit within the context of adult learning theory in that they highlight the need for tailored technology orientation sessions in launching remotely delivered exercise programs for older adults. In addition, the interperson variability in technical challenges during orientation and the first 2 weeks of classes lends further evidence for an individualized approach to technical support. This may pose a challenge for programs regarding sufficient funding for ongoing tailored technical support. Therefore, further research is needed to better quantify the return on the investment for tailored technology orientations in engaging and retaining older adults in remotely delivered health promotion programs.

Most participants in the study had access to technology and the internet. Although more than half of the participants (23/44, 52%) were a little or somewhat confident in their ability to go on the web, the scores for anxiety related to using technology were relatively low. However, participants with low confidence to go on the web had lower gerontechnology anxiety scores than those who were somewhat confident going on the web. This may be a reflection of those with little confidence having less experience with technology overall, including situations that they could not manage or overcome. It is worth noting that the STAM questionnaires assess technology acceptance in a general sense [34]. Participants in the study knew that they would need to use technology for a specific purpose (ie, accessing group exercise classes) and one that clearly had value, as indicated by the interviews. The knowledge that technology use would be targeted and specific, rather than technology use in general, may have impacted their acceptance scores on the questionnaire. The overall high scores for attitudinal beliefs align with the interview responses that technology would be useful and effective to access the classes they regarded as valuable. The high scores for control beliefs also align with the participants’ responses in the interviews. The control belief questions measure confidence in being skillful at using technology and using technology after being instructed on how to do it. High scores on these questions correspond with the interview themes that opportunities to participate in high-value activities motivate willingness to persevere through the technology concerns and belief in the ability to learn new things supersedes technology-related frustration.

There were relatively few hardware or internet issues after the classes began, in part, because the study staff conducted the one-on-one technology needs assessment before the initial orientation meeting. Orientation to the Zoom features was a key part of the initial instruction, resulting in most participants not requiring additional support thereafter. Overall, the rates of the technical challenges were low at the onset of remote Enhance Fitness classes and declined further after the first 2 weeks of classes. The most common challenges addressed during the orientation session were related to camera adjustments to allow for full body view while exercising at a distance sufficient to see and hear the instructor. During the first 2 weeks of classes, the most common problem, experienced at a rate of 10%, was joining or staying on the videoconference. By the third week of classes, joining or staying on the videoconference remained the most common challenge but decreased to a rate of less than 3%. We attribute the relatively low rates of technical challenges to (1) a sample of relatively educated older adults including few with previous videoconference experience, (2) the individualized orientation session conducted before the start of classes with the opportunity for individual follow-up orientation sessions as needed, (3) relatively immediate implementation of tele–Enhance Fitness classes after the technology orientation combined with the class structure (ie, 3 days per week) for frequent repetition of the technology procedures, and (4) standby technical assistance before and during the tele–Enhance Fitness classes. Consideration of these factors, which align with adult learning theory principles, may be beneficial in a successful launch of a remote exercise program.

Owing to the limited research to date on adapting an evidence-based exercise program for internet-based delivery to older adults, the mixed-method approach provides advantages at the current stage. Although previous studies have examined technology adoption in older adults and learning strategies for teaching older adults on technology use [44,45], few studies have focused on strategies for supporting older adults to participate in tele-exercise programs. Using the exploratory sequential mixed-method design allowed participant preferences to be incorporated into the technology orientation sessions and then quantified the challenges encountered with and attendance to the exercise classes, an indicator of the success of technology adoption. With the goal of expanding access to tele-exercise classes, an imperative next step is to replicate the study in a larger and more diverse population of older adults, especially regarding technology experience and gerontechnology anxiety. An iterative process will help to refine the best practices in supporting older adults in tele-exercise participation.

### Limitations

We acknowledge the limitations of this study. Computer systems and software require frequent updates and occasionally undergo format changes. In this study, we did not examine how these updates or format changes impact perceptions of tele-exercise or the challenges encountered in managing software updates or new technology additions. The study sample consisted predominantly of White, college-educated women with relatively low gerontechnology anxiety and a history of technology use. Therefore, the findings cannot be generalized to all older adults. This study does not allow us to predict how technology barriers will be perceived in more diverse populations. For example, the findings related to the theme of belief in the ability to learn new things may be more relevant to our homogeneous sample of more educated White women and cannot be generalized to all older adults. The current population was originally recruited from community-based senior centers and for the interview sample, had previously participated in the in-person Enhance Fitness classes. Purposeful sampling of Enhance Fitness participants allowed us to capture a range of perspectives from people with varying levels of comfort in using technology to access group exercise classes. We intentionally interviewed older adults who had experience with in-person exercise classes to understand their needs in transitioning to remote classes. We acknowledge that their perceptions of the benefits of the classes may have enhanced their desire to learn and use technology. However, the high attendance rates in the full sample, including those with and without prior experience with in-person Enhance Fitness, provides some evidence that people without prior in-person Enhance Fitness exercise experience can overcome technology barriers to engage in remote exercise classes, if provided with sufficient support.

### Implications and Conclusions

The COVID-19 pandemic has resulted in extreme measures to reduce the spread of the virus, including the cessation of community-based programs for older adults in senior centers and fitness facilities. The loss of access to these programs impacts social opportunities, management of chronic conditions, and fall risk in older adults nationwide. With the availability of vaccines, there is an expectation of returning to community programs in the near future. However, it is too early to predict how long the physical distancing measures will need to be followed, and therefore how long until older adults can safely resume participating in community-based exercise programs. Until that time, remotely delivered programs remain a viable option and, for some, a preferred mode of delivery because of its convenience. On the basis of our findings, with appropriate support and attention, older adults are able to participate in remote exercise using technology. Importantly, these findings are also relevant to the millions of older adults who do not have access to in-person community programs, such as those who are homebound or living in rural areas. With sufficient tailoring of technology and support to meet the needs of diverse populations, continued development of best practices in this area has the potential to allow previously hard-to-reach populations of older adults to participate in health-enhancing, evidence-based exercise programs.

## Appendix

Multimedia Appendix 1 Semistructured interview guide.

## Abbreviations

STAM: Senior Technology Acceptance Model
