# Supplementary material for: Technology Support Challenges and Recommendations for Adapting an Evidence-Based Exercise Program for Remote Delivery to Older Adults: Exploratory Mixed Methods Study
Source: JMIR Aging. 2021 Dec 9;4(4):e27645. doi: 10.2196/27645 (PMC8704113; doi:10.2196/27645)
Supplement: Multimedia Appendix 1 [file aging_v4i4e27645_app1.docx]

**Multimedia Appendix 1. Semistructured interview guide.**

**Interview Guide to Assess Study Participant Needs and Concerns for Remotely Delivered Classes**

What concerns do you have about transitioning from in-person classes to a virtual version?

What would make this transition easier for you?

*Prompt: What support can the research staff provide to make this process easier?*

How do you feel about using some of your own technology and Wi-Fi to participate in videoconferencing classes?

What is most helpful when you first try using a new technology? For example, do you prefer to figure it out for yourself, no matter how long it takes, or do you prefer having someone walk you through step by step.

What has been less helpful in the past for using new technology?

What is frustrating to you when trying new technology, and what have you done to help prevent that frustration?

What can we do to best support you when challenges occur? (What would help to minimize any stress or frustration as we work through new challenges?)

What videoconference platforms have you used previously (zoom, facebook group, Teams, google, etc). Do you have any preferences among the ones you have tried? Are there features of any of those programs that you like in particular or dislike in particular?

What are your recommendations for adding time or opportunity for socializing? *Any preference for time added before or after each class? What would entice you to participate in the additional social opportunities outside of the class time? Would you like a platform that enables you to “chat” with other class participants, instructors, or study staff?*

What would help you to participate in the virtual EF classes? (Prompts: *Would* *one-on-one set-up and practice sessions be helpful? How about access to videos to learn or practice the exercises outside of class time? Someone from the research staff to monitor your exercise or troubleshoot any technology challenges?*

Do you have concerns about exercising at home [physical space, privacy]?

Do you have concerns about exercising at home without another person around?

Do you have a location in your home where you feel comfortable participating in the online classes? Is there anything we can do to help you identify a safe space to exercise and participate in the educational class discussions?
